# A novel system to provide information via online YouTube videos and an evaluation of current online information about hereditary breast cancer

**DOI:** 10.1007/s12282-023-01512-y

**Published:** 2023-11-23

**Authors:** Asumi Iesato, Atsushi Fushimi, Rie Tahara, Mitsuo Terada, Madoka Iwase, Chihiro Kawamura, Nami Yamashita

**Affiliations:** 1 General Incorporated Association BC Tube, 1-5-6 Kudan-minami, Chiyoda-ku, Tokyo, 102-0074 Japan; 2 NEXT-Ganken Program, Cancer Cell Diversity Project, Japanese Foundation for Cancer Research, 3-8-31, Ariake, Koto-ku, Tokyo, 135-8550 Japan; 3 Department of Surgery, The Jikei University School of Medicine, 3-25-8 Nishi-Shinbashi, Minato-ku, Tokyo, 105-8461 Japan; 4 Department of Breast Surgery, Nagoya City University Graduate School of Medical Sciences, 1-Kawasumi, Mizuho-cho, Mizuho-ku, Nagoya, Aichi 467-8602 Japan; 5 Department of Breast and Endocrine Surgery, Nagoya University Hospital, 65 Tsurumai-cho, Showa-ku, Nagoya, Aichi 466-8560 Japan; 6 Breast Oncology Center, Cancer Institute Hospital of Japanese Foundation for Cancer Research, 3-8-31, Ariake, Koto-ku, Tokyo, 135-8550 Japan

**Keywords:** Hereditary breast cancer, YouTube, Online health information, Peer review, Patient and public involvement (PPI)

## Abstract

**Background:**

The internet, especially YouTube, has become a prominent source of health information. However, the quality and accuracy of medical content on YouTube vary, posing concerns about misinformation. This study focuses on providing reliable information about hereditary breast cancer on YouTube, given its importance for decision-making among patients and families. The study examines the quality and accessibility of such content in Japanese, where limited research has been conducted.

**Methods:**

A nonprofit organization called BC Tube was established in May 2020 to create informative videos about breast cancer. The study analyzed 85 YouTube videos selected using the Japanese keywords “hereditary breast cancer” and “HBOC”, categorized into six groups based on the source of upload: BC Tube, hospitals/governments, individual physicians, public-interest organizations/companies, breast cancer survivors, and others. The videos were evaluated based on various factors, including content length, view counts, likes, comments, and the presence of advertisements. The content was evaluated using the PEMAT and DISCERN quality criteria.

**Results:**

BC Tube created high-quality videos with high scores on PEMAT understandability, significantly outperforming other sources. Videos from public-interest organizations/companies received the most views and likes, despite their lower quality. Videos from medical institutions and governments were of superior quality but attracted less attention.

**Conclusions:**

Our study emphasizes the importance of promoting accessible, easy-to-understand, and widely recognized medical information online. The popularity of videos does not always correspond to their quality, emphasizing the importance of quality evaluation. BC Tube provides a peer-reviewed platform to disseminate high-quality health information. We need to develop high-quality online health information and encourage the promotion of evidence-based information on YouTube.

## Introduction

With advanced information technology, the internet has become an important and easily accessible source of health information for patients [1]. Online video-sharing platforms are popular due to lifestyle changes resulting from the increased use of smartphones, tablets, social media, and cloud computing [2]. YouTube is the most widely used online video-sharing platform (www.youtube.com; YouTube LLC, San Bruno, CA) [3, 4]. The impact of YouTube on the dissemination of health information, including information about breast cancer, to the general public is continuously being studied [5–7]. Video can assist patients with low literacy skills and aid in making complex decisions by providing visual information. Videos may be more appealing to younger patients accustomed to multimedia as a primary source of information [8]. Medical information is fundamental to decision-making among cancer patients and families [9]. Unfortunately, a significant amount of internet medical resources is unregulated and may contain inaccurate or misleading information [10]. The dissemination of biased and misinformative medical content is a major societal issue with serious implications for decision-making and outcomes [11]. The quality of health information depends not only on accuracy and reliability but also on website interactivity and usability, including vested commercial or financial interests of the authors or sponsors and characteristics of the authors and language [12]. Quality, easy-to-understand, and accessible information is needed.

Hereditary breast cancer accounts for 5–10% of all breast cancers [13], and hereditary breast and ovarian cancer (HBOC) is the most common type [14]. *BRCA1/2* genetic testing and medical management, including prophylactic surgery and surveillance of HBOC patients, have been covered by Japanese national health insurance since April 2020, leading to increased *BRCA1/2* genetic testing and prophylactic interventions [15, 16]. The clinical benefits of genetic testing and therapeutic and preventive interventions can be optimized by providing appropriate information on hereditary breast cancer to patients and their families. An online video-sharing platform is practical for providing hereditary breast cancer information because information can be delivered to a wide audience, including patients, relatives, and people who have not yet developed cancer, and videos can be viewed repeatedly. However, few studies have examined the hereditary breast cancer information available on YouTube [5], and no studies have focused on the distribution of YouTube videos in Japanese.

In May 2020, we established an editorial board for a nonprofit general incorporated association called Breast Cancer Tube (BC Tube). We began providing animated, easy-to-understand videos with reliable medical information about breasts and breast cancer via the YouTube channel “Breast Cancer Encyclopedia [BC Tube Editors].” The purpose of the videos was to promote public breast awareness and create an internet learning environment where patients and families can access quality internet resources for breast cancer. The advantage of online video-sharing platforms is the ability to provide information on hereditary breast cancer to a wide audience, including patients, families, distant relatives who cannot visit the doctor, and people who do not have the opportunity to visit a medical institution. Moreover, videos can be viewed repeatedly and free of charge. Multimedia delivery of information using videos, animations, infographics, text, and audio can potentially enhance comprehension [17, 18]. However, the usefulness of our content compared to other videos was unclear.

The aim of this study was to present our online video production activities and assess the quality, content, and readability of current health information about hereditary breast cancer on YouTube. Using the following search terms, “hereditary breast cancer (kanji and hiragana notation)” and “HBOC,” we evaluated the first 100 YouTube videos and conducted a systematic evaluation of the quality of breast cancer information available on YouTube using validated assessment instruments, including the Patient Education Materials Assessment Tool (PEMAT) [19] and the DISCERN quality criteria for consumer health information [20]. Furthermore, we compared the quality of BC Tube content with other online videos.

## Materials and methods

### Nonprofit general incorporated association called BC Tube

We established a nonprofit general incorporated association called BC Tube in May 2020. Up to March 31st, 2023, 52 videos were created, including videos on breast health education (symptoms, breast awareness, and epidemiology), screening, diagnosis, treatment of early-stage breast cancer, metastatic/recurrent breast cancer, side effects of treatment, and hereditary breast cancer. This content was viewed over 1,200,000 times over 59,000 h per year from July 2020 to March 2023. Five of our videos were about hereditary breast cancer.

### Searching and selection of YouTube videos for study inclusion

To examine the current information about hereditary breast cancer and HBOC on YouTube, we performed searches using the keywords “hereditary breast cancer (“cancer” written in kanji)” and “hereditary breast cancer (“cancer” in Hiragana), and “HBOC” in Japanese on July 7th, 2022 and selected the top 100 YouTube videos for each search term. The searches were conducted after cleaning the search history and without logging into any account. We used relevance-based ranking to sort the results, and the top 100 videos were assessed. The number of videos analyzed was based on previous studies that analyzed the first 50–170 videos [21–24].

Two non-medical investigators independently screened the videos. The independent investigators had no conflicts of interest with BC Tube or any other YouTube content creation organizations. The review included Japanese language videos and English videos with Japanese subtitles. We excluded duplicate videos and content not containing information about hereditary breast cancer.

### Video classification

To compare the distinctions among sources and assess the contrasts between BC Tube and the others, videos were classified by the source of upload into 6 groups, including BC Tube, hospitals/governments, individual physicians, public-interest organizations/companies, breast cancer survivors, and others. The hospital/governments group included videos by the national cancer institute, prefectures, universities, and hospitals/clinics (single facility). The public-interest organizations/companies included broadcasting stations, newspapers, nonprofit organizations, and profit organizations. The “others” group included three recorded deliveries of other distributors’ videos and three videos by unknown sources. All three recorded deliveries were from public-interest organizations/companies.

### Viewing status and quality of content analyses

The source/providers of content, the time since upload, the content length, the view counts, the number of comments, the number of likes, and advertisement displays were recorded. The quality of breast cancer information was evaluated systematically with a range of metrics, including content accuracy, readability, and accountability, using validated assessment instruments, including the PEMAT [25] and the DISCERN quality criteria for consumer health information [26], as shown in Table 2. PEMAT is a systematic method to evaluate the understandability and actionability of patient education materials. Separate tools are available for print and audiovisual materials. We used the PEMAT instrument for audiovisual materials. In the DISCERN score, Questions 1–8 focus on the reliability of the contents to judge whether it can be trusted as a source of information about treatment choices. Questions 9–15 focus on specific details about treatment choices. Question 16 shows the overall quality rating at the end of the instrument. Two independent investigators evaluated the content independently. We compared the quality of the content from the six sources.

### Statistical analysis

Statistical analysis was performed using R software and GraphPad Prism 8 software. One-way analysis of variance for multiple comparison tests. Data are reported as the averages with standard errors of the average for each group. Results were considered significant at *p* values below 0.05.

## Results

### The process of creating videos by the BC Tube

**Fig. 1:**
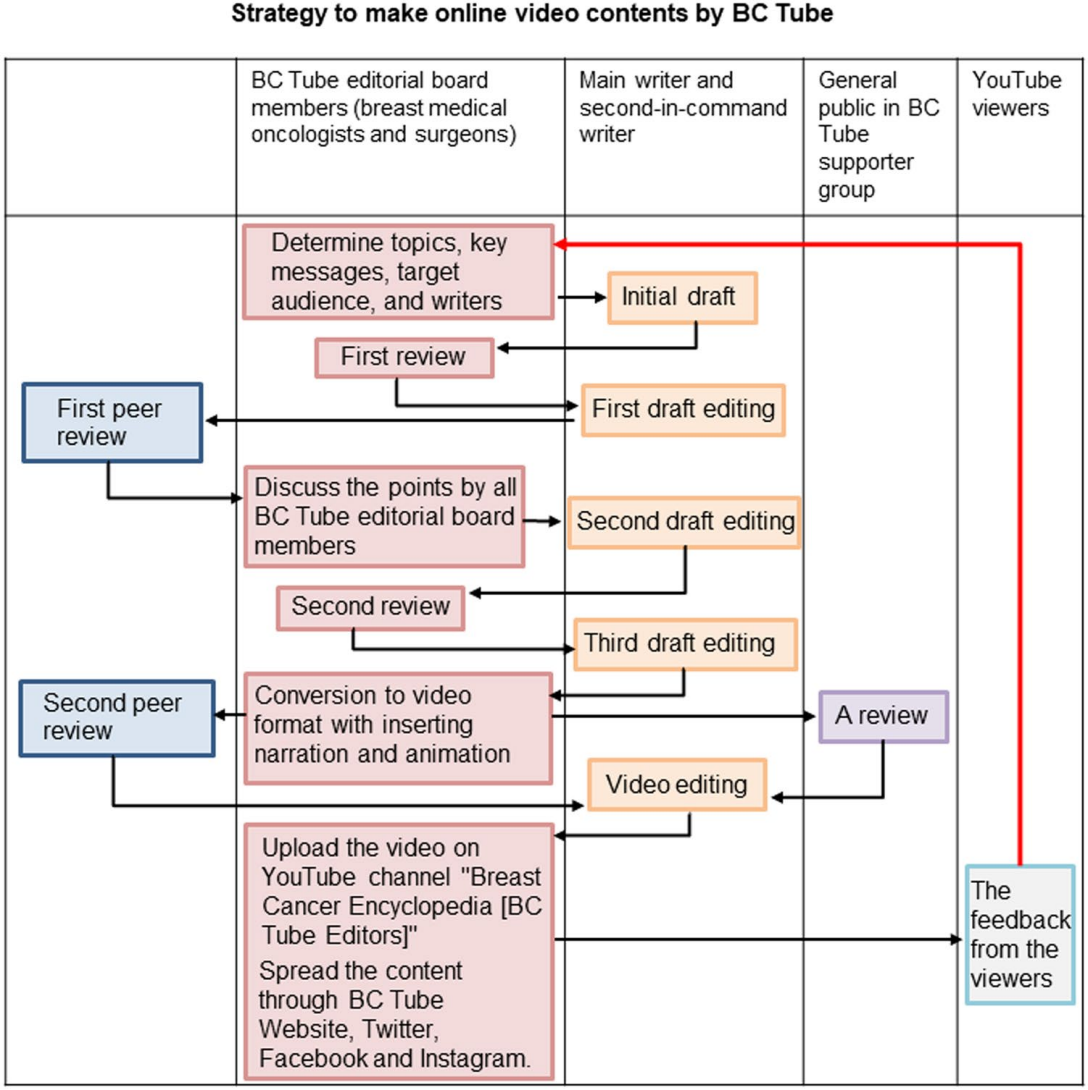
Strategies for creating online video content by BC Tube

We created online video content on breast cancer (Fig. 1). Content development was discussed by multiple breast medical oncologists and surgeons. First, the key messages and target audience were discussed among the BC Tube editorial board members. Then, the main writer and the second-in-command staff were selected. The BC Tube editorial board reviewed the draft. If the BC Tube editorial board members agreed that the content was well-produced, the content was reviewed by an external peer review group of independent breast medical oncologists and surgeons to ensure scientific validity. The narration text was also reviewed. After external peer review, the revised content was reviewed by all BC Tube editorial board members again. The content was converted to a video format with narration and animation. The narration text was inserted at the bottom of the video. Videos were no more than 10 min to promote sustained concentration while viewing the YouTube viewing screen. The video was reviewed by the BC Tube Support Group to ensure readability and ensure that no offensive expressions were included. The BC Tube Support Group is a general citizen group consisting of 110 volunteers who were selected through open recruitment, including women and men who have not been diagnosed with breast cancer, breast cancer patients, breast cancer survivors, and medical staff other than breast medical oncologists/surgeons.

Finally, the videos were uploaded to the YouTube channel “Breast Cancer Encyclopedia [BC Tube Editors]” (https://www.youtube.com/@-BCTube), and the content was spread through social media, including the BC Tube Website, Twitter, Facebook, and Instagram. A link to a questionnaire form was placed in the summary section of the YouTube video to obtain opinions and feedback from viewers.

### Content characteristics

**Fig. 2:**
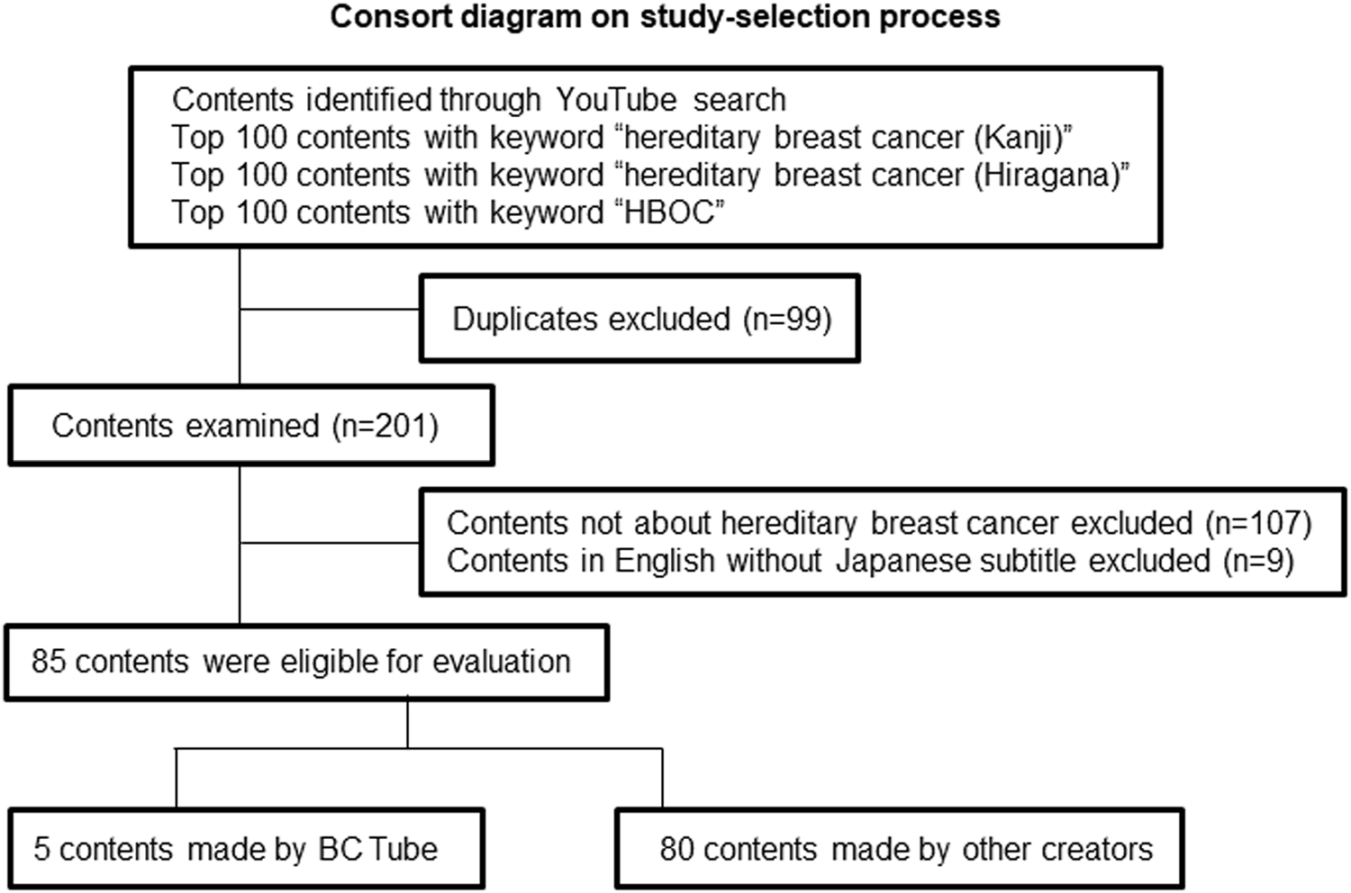
Consort diagram on study-selection process

The strategy to select the videos included in the analysis is outlined in the CONSORT diagram (Fig. 2). Ninety-nine duplicated videos were excluded, and 107 videos without information about hereditary breast cancer were excluded, including content with general information about breast cancer treatment, introduction to statistical analysis using genetic breast cancer data, and content introducing cars with HBOC in the name. Nine videos in English without any Japanese information were excluded. Thus, 85 videos were eligible for this study.

**Fig. 3:**
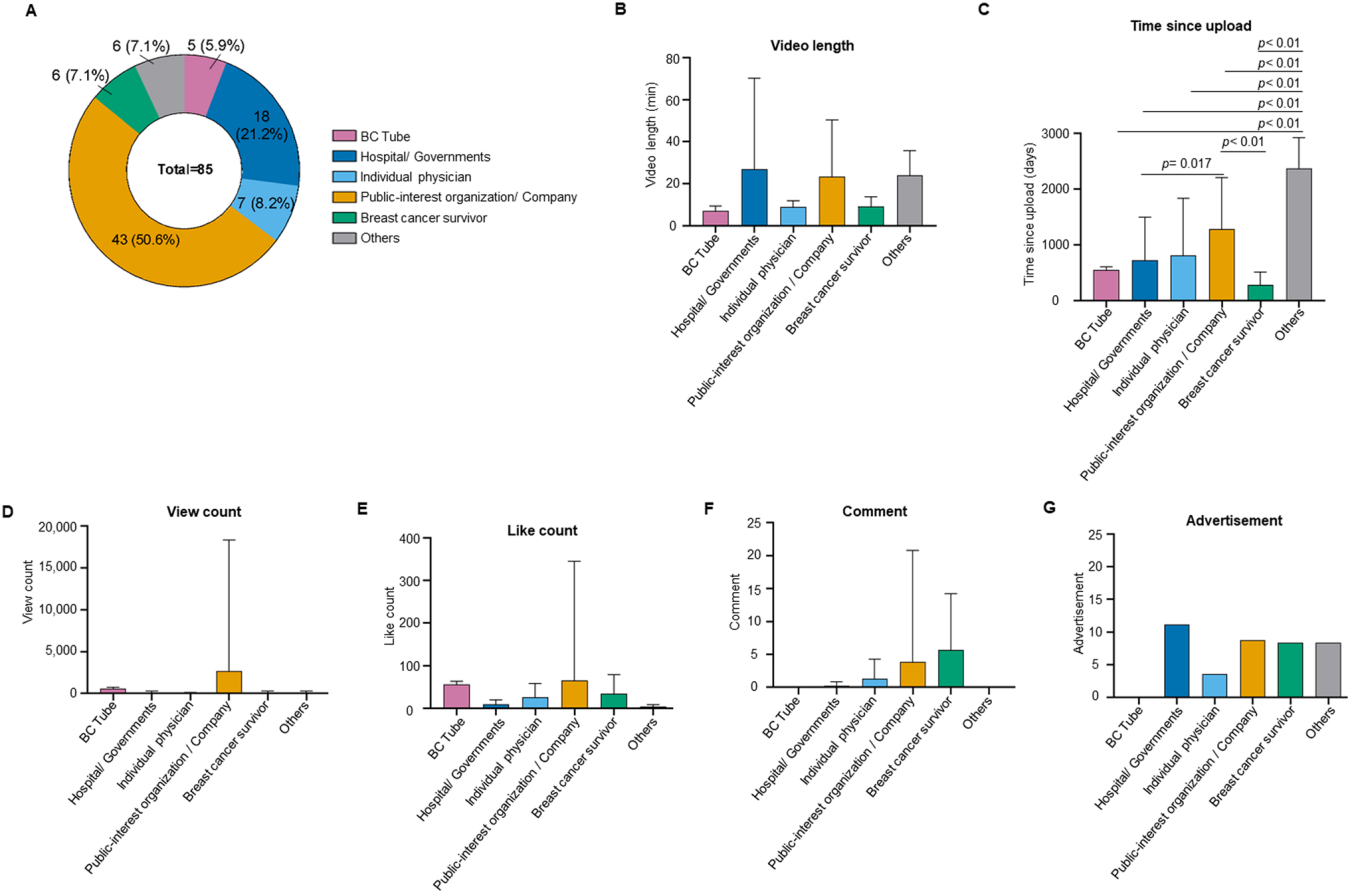
Characteristics of online video content by creator

A majority of the videos (43 videos, 50.6%) were provided by public-interest organizations/companies, followed by hospitals/governments (18 videos, 21.2%), individual physicians (7 videos, 8.2%), breast cancer survivors (6 videos, 7.1%), and others (6 videos, 7.1%) (Fig. 3A, Table 1). Five videos about genetics and breast cancer produced by the BC Tube were included in the top 100 for “hereditary breast cancer (in hiragana)”; 4 videos were included in the top 100 for “hereditary breast cancer (in kanji),” and 2 videos were included in the top 100 for “HBOC.”

**Table 1:** Number and characteristics of online video content by creator

	BC Tube	Hospital/Governments	Individual physician	Public-interest organization/company	Breast cancer survivor	Others
N of Contents in top 100 search	5	18	7	43	6	6
Video length (minutes)	7.1 ± 2.3	26.9 ± 43.3	8.9 ± 3.0	23.3 ± 27.0	9.0 ± 4.7	24.0 ± 11.7
Time since upload (days)	552.4 ± 52.0	727.7 ± 771.2	812.9 ± 1023.9	1288.6 ± 913.8	283.0 ± 226.4	2374.0 ± 551.5
View counts (times)	5614.8 ± 1690.2	1115.7 ± 1307.7	540.4 ± 395.6	26,686.8 ± 156,763.4	1343.7 ± 1572.3	1213.0 ± 1530.2
Like counts (times)	56.2 ± 6.9	9.4 ± 9.6	26.0 ± 31.9	65.0 ± 280.1	34.2 ± 44.8	3.5 ± 4.4
Comments (times)	NA	0.2 ± 0.6	1.3 ± 3.0	3.8 ± 17.0	5.7 ± 8.5	0
Advertisement *N* (%)	0 (0)	8 (44.4)	1 (14.3)	15 (34.9)	2 (33.3)	2 (33.3)

The average length of the BC Tube (7.1 ± 2.3 min), individual physician (8.9 ± 3.0 min), and breast cancer survivor (9.0 ± 4.7 min) videos tended to be shorter compared with the length of the hospital/government (26.9 ± 43.3), public-interest organization/company (23.3 ± 27.0 min), and “other” (24.0 ± 11.7 min) videos. However, differences between groups were not significant (Fig. 3B, Table 1). The time since the upload of videos on YouTube was significantly longer for videos posted by public-interest organizations/companies compared with videos posted by hospitals/governments (*p* = 0.017) and breast cancer survivors (*p* < 0.01) (Fig. 3C, Table 1). The time since upload of videos from the “others” group was longer than the times for other groups (Fig. 3C, Table 1). These results suggest that public-interest organizations and companies focused earlier on providing information on hereditary breast cancer via YouTube than other sources. The same trend was evident in the “others” group, which included three recorded videos from public-interest organizations. Most videos created by hospitals and governments and breast cancer survivors were sent out around 2020, when health insurance coverage for *BRCA1/2* genetic testing and HBOC treatment began in Japan.

We used YouTube Analytics to check the viewing status of BC Tube videos. The view counts per day decreased after the videos were posted. Therefore, we compared the total view counts instead of the number of views per unit of time. Public-interest organizations/companies exhibited the highest number of views (26,686.8 ± 156,763.4), followed by BC Tube (5614.8 ± 1690.2) (Fig. 3D, Table 1). Hospital/ governments (1115.7 ± 1307.7), breast cancer survivors (1343.7 ± 1572.3), and the “others” group (1213.0 ± 1530.2) showed similar view counts whereas individual physicians (540.5 ± 395.6) showed the lowest view counts although the differences were not significant (Fig. 3D, Table 1). Three videos from hospitals/governments and 10 videos from public-interest organizations/companies disabled the view counters on YouTube. Public-interest organizations/companies (65.0 ± 280.1) had the highest number of “likes,” followed by BC Tube (56.2 ± 6.9) and breast cancer survivors (34.2 ± 44.8) (Fig. 3E, Table 1).

All BC Tube videos turned off the comments to avoid personal consultations but employed a survey form in the summary section to receive feedback. Five BC Tube videos, 8 videos from hospitals/governments, and 15 videos from public-interest organizations/companies disabled comments. Excluding these videos, breast cancer survivors had the highest number of comments (5.7 ± 8.5) followed by public-interest organizations/companies (3.8 ± 17.0); however, the differences were not significant (Fig. 3F, Table 1). The viewers may find assessing the impartiality of content creators who display advertisements on YouTube difficult. To address this issue, the BC Tube does not show advertisements. Aside from the BC Tube, no significant differences in the number of videos with advertisements among the four groups were detected (Fig. 3G, Table 1).

### Quality analysis of YouTube content

**Fig. 4:**
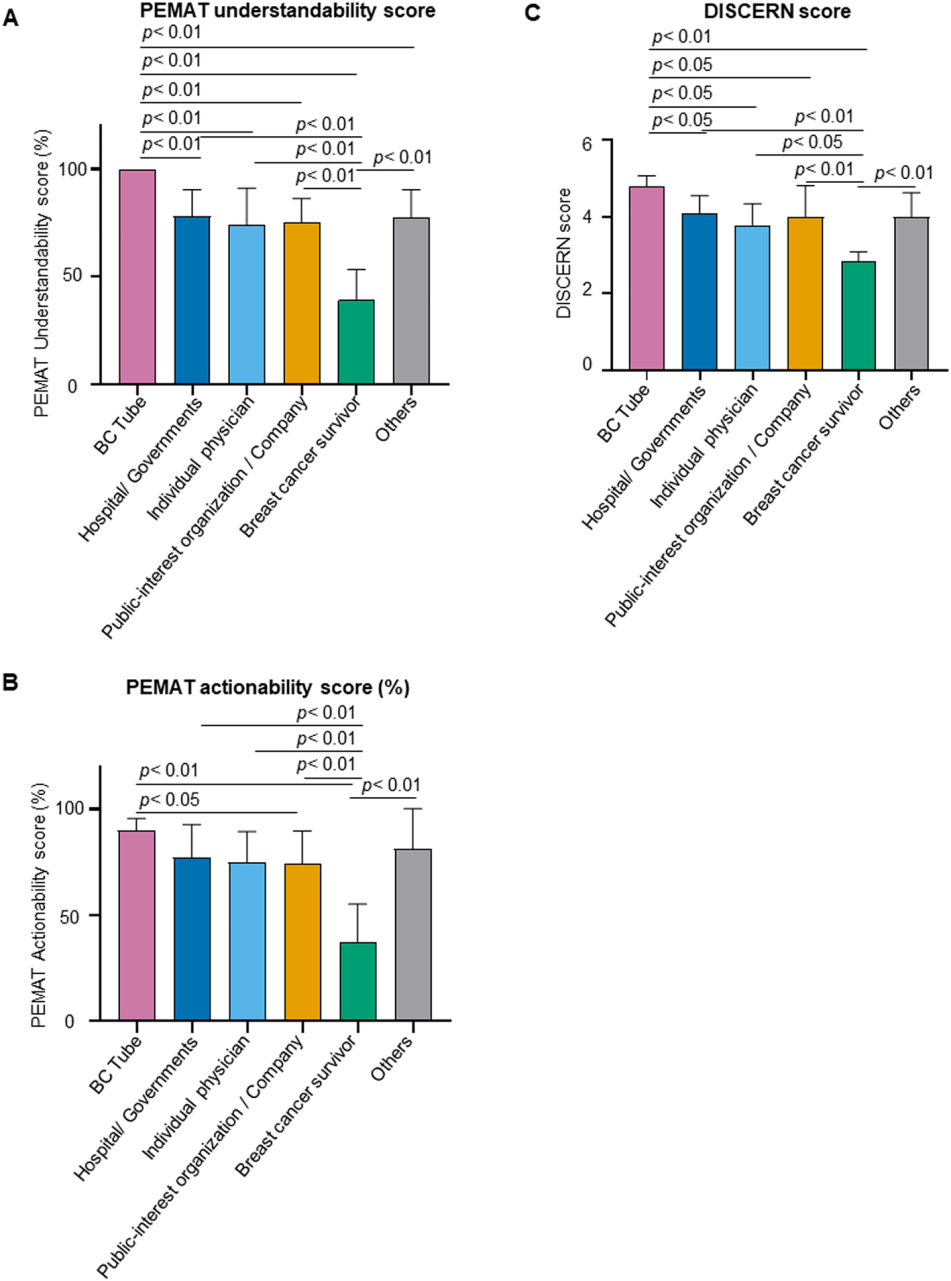
PEMAT understandability score

To elucidate the quality of hereditary breast cancer information content, we applied two validated assessment instruments: PEMAT and DISCERN quality criteria. All 5 videos made by the BC Tube had perfect scores for PEMAT understandability scores; the PEMAT scores for BC Tube videos were significantly higher than the scores of the other groups (Fig. 4A). The videos from breast cancer survivors had significantly lower PEMAT understandability scores compared with the other groups (Fig. 4A). Videos from BC Tube, hospitals/governments, individual physician, public-interest organizations/companies and “others” had significantly higher PEMAT actionability scores compared with breast cancer survivors (*p* < 0.01 in all) (Fig. 4B). Videos made by the BC Tube had higher PEMAT actionability scores than public-interest organizations/companies (*p* < 0.05) (Fig. 4B).

Question 16 of the DISCERN score is an overall rating of the content quality. The BC Tube videos had higher overall quality ratings compared with all other groups (Fig. 4C). The breast cancer survivor videos had significantly lower DISCERN score compared with the other groups (Fig. 4C). Collectively, these results indicate that videos from BC Tube, hospitals, and governments were high quality, leading viewers to take action. Videos sent by individual contributors, such as breast cancer survivors, were generally of lower quality based on the qualitative scale (Tables 2 and 3).

**Table 2:** Evaluation criteria for quality assessment using PEMAT and DISCERN

PEMAT instrument		
Domain: understandability		
Q1	The material makes its purpose completely evident	Disagree = 0, Agree = 1
Q3	The material uses common, everyday language	Disagree = 0, Agree = 1
Q4	Medical terms are used only to familiarize audience with the terms. When used, medical terms are defined	Disagree = 0, Agree = 1
Q5	The material uses the active voice	Disagree = 0, Agree = 1
Q8	The material breaks or “chunks” information into short sections	Disagree = 0, Agree = 1, Very short material = N/A
Q9	The material’s sections have informative headers	Disagree = 0, Agree = 1, Very short material = N/A
Q10	The material presents information in a logical sequence	Disagree = 0, Agree = 1
Q11	The material provides a summary	Disagree = 0, Agree = 1, Very short material = N/A
Q12	The material uses visual cues (e.g., arrows, boxes, bullets, bold, larger font, highlighting) to draw attention to key points	Disagree = 0, Agree = 1, Very short material = N/A
Q13	Text on the screen is easy to read	Disagree = 0, Agree = 1, No text = N/A
Q14	The material allows the user to hear the words clearly (e.g., not too fast, not garbled)	Disagree = 0, Agree = 1, No narration = N/A
Q18	The material uses illustrations and photographs that are clear and uncluttered	Disagree = 0, Agree = 1, No visual aids = N/A
Q19	The material uses simple tables with short and clear row and column headings	Disagree = 0, Agree = 1, No tables = N/A
Understandability score (%)		(Total points/total possible points × 100)
Domain: actionability		
Q20	The material clearly identifies at least one action the user can take	Disagree = 0, Agree = 1
Q21	The material addresses the user directly when describing actions	Disagree = 0, Agree = 1
Q22	The material breaks down any action into manageable, explicit steps	Disagree = 0, Agree = 1
Q25	The material explains how to use the charts, graphs, tables, or diagrams to take actions	Disagree = 0, Agree = 1, No charts, graphs, tables, diagrams = N/A
Actionability score (%)		(Total points/total possible points × 100)

DISCERN instrument		Rating
Q1	Are the aims clear?	1:no, 2–4:partially, 5:yes. If the answer is ‘no’, go directly to Q3
Q2	Does it achieve its aims?	1:no, 2–4:partially, 5:yes
Q3	Is it relevant?	1:no, 2–4:partially, 5:yes
Q4	Is it clear what sources of information were used to compile the publication (other than the author or producer)?	1:no, 2–4:partially, 5:yes
Q5	Is it clear when the information used or reported in the publication was produced?	1:no, 2–4:partially, 5:yes
Q6	Is it balanced and unbiased?	1:no, 2–4:partially, 5:yes
Q7	Does it provide details of additional sources of support and information?	1:no, 2–4:partially, 5:yes
Q8	Does it refer to areas of uncertainty?	1:no, 2–4:partially, 5:yes
Q9	Does it describe how each treatment works?	1:no, 2–4:partially, 5:yes
Q10	Does it describe the benefits of each treatment?	1:no, 2–4:partially, 5:yes
Q11	Does it describe the risks of each treatment?	1:no, 2–4:partially, 5:yes
Q12	Does it describe what would happen if no treatment is used?	1:no, 2–4:partially, 5:yes
Q13	Does it describe how the treatment choices affect overall quality of life?	1:no, 2–4:partially, 5:yes
Q14	Is it clear that there may be more than one possible treatment choice?	1:no, 2–4:partially, 5:yes
Q15	Does it provide support for shared decision-making?	1:no, 2–4:partially, 5:yes
Q16	Based on the answers to all of the above Qs, rate the overall quality of the publication as a source of information about treatment choices	1:serious or extensive shortcomings, 2–4:potentially important but not serious shortcomings, 5:minimal shortcomings

**Table 3:** Quality assessment results of online video content using PEMAT and DISCERN

	BC tube		Hospital/governments		Individual physician		Public-interest organization/company		Breast cancer survivor		Others		
	Mean	SD	Mean	SD	Mean	SD	Mean	SD	Mean	SD	Mean	SD	*p* value
DISCERN													
Q1	5.0	0.0	4.2	1.0	4.6	0.8	4.2	1.0	2.8	0.4	4.2	1.3	0.01
Q2	5.0	0.0	4.3	1.0	4.1	1.1	3.9	1.3	2.8	0.4	4.2	1.3	0.05
Q3	5.0	0.0	4.3	0.8	4.3	1.0	4.3	1.0	3.5	1.0	4.7	0.5	0.14
Q4	5.0	0.0	2.9	1.1	2.7	1.0	3.0	1.3	1.3	0.5	3.3	1.4	0.00
Q5	5.0	0.0	2.3	1.2	1.9	0.4	2.3	1.1	1.5	0.5	2.3	1.0	0.00
Q6	4.4	0.5	3.4	0.7	3.3	0.8	3.0	1.0	1.8	0.4	3.0	1.3	0.00
Q7	4.6	0.5	3.6	0.9	3.1	0.7	3.0	1.1	1.7	0.8	3.3	1.5	0.00
Q8	4.2	0.8	3.1	0.7	3.1	1.1	3.1	1.0	1.5	0.5	3.2	1.3	0.00
Q9	4.2	0.4	4.2	0.7	4.0	0.8	3.9	1.2	2.5	1.4	4.3	1.6	0.04
Q10	4.6	0.9	4.3	0.8	3.6	0.8	3.9	1.2	3.0	1.3	4.3	1.6	0.10
Q11	4.4	0.9	3.6	0.9	3.1	1.2	3.1	1.1	2.2	1.0	3.0	1.3	0.02
Q12	4.0	0.7	3.0	1.1	2.7	0.8	2.8	1.2	1.5	0.5	2.5	0.8	0.01
Q13	4.6	0.9	4.2	0.7	3.9	0.9	4.1	1.1	3.2	1.5	3.8	1.5	0.30
Q14	4.2	1.1	3.6	0.9	3.1	1.1	3.2	1.2	2.0	0.9	3.0	1.3	0.03
Q15	5.0	0.0	3.9	1.0	3.3	0.8	3.7	1.2	2.2	1.2	4.0	1.7	0.00
Q16	4.6	0.5	3.4	0.7	2.9	0.9	3.3	1.0	1.7	0.5	3.2	1.3	0.00
PEMAT													
Q1	1.0	0.0	0.8	0.4	0.7	0.5	0.7	0.5	0.2	0.4	0.7	0.5	0.06
Q3	1.0	0.0	0.9	0.2	1.0	0.0	1.0	0.2	0.8	0.4	1.0	0.0	0.55
Q4	1.0	0.0	0.9	0.3	1.0	0.0	1.0	0.2	0.8	0.4	1.0	0.0	0.43
Q5	1.0	0.0	0.9	0.3	1.0	0.0	0.7	0.5	0.0	0.0	0.8	0.4	0.00
Q8	1.0	0.0	0.2	0.4	0.0	0.0	0.0	0.0	0.0	0.0	0.0	0.0	0.00
Q9	1.0	0.0	0.4	0.5	0.3	0.5	0.3	0.5	0.0	0.0	0.5	0.5	0.01
Q10	1.0	0.0	0.2	0.4	0.1	0.4	0.2	0.4	0.0	0.0	0.3	0.5	0.00
Q11	1.0	0.0	0.5	0.5	0.3	0.5	0.6	0.5	0.2	0.4	0.7	0.5	0.06
Q12	1.0	0.0	0.5	0.5	0.3	0.5	0.6	0.5	0.2	0.4	0.7	0.5	0.06
Q13	1.0	0.0	0.8	0.4	0.9	0.4	0.7	0.5	0.7	0.5	0.4	0.5	0.37
Q14	1.0	0.0	0.9	0.2	1.0	0.0	0.9	0.3	1.0	0.0	1.0	0.0	0.91
Q18	1.0	0.0	0.5	0.5	0.4	0.5	0.6	0.5	0.0	0.0	0.8	0.4	0.01
Q19	1.0	0.0	0.8	0.4	0.5	0.6	0.7	0.5	0.0	0.0	0.8	0.4	0.01
PEMAT understandability score (%)	100.0	0.0	62.5	20.5	60.3	21.9	61.2	21.0	28.0	17.2	65.3	23.9	0.00
Q20	1.0	0.0	1.0	0.0	0.9	0.4	0.9	0.4	0.7	0.5	0.8	0.4	0.32
Q21	1.0	0.0	0.8	0.4	1.0	0.0	0.6	0.5	0.5	0.5	0.7	0.5	0.05
Q22	0.6	0.5	0.5	0.5	0.1	0.4	0.3	0.5	0.0	0.0	0.5	0.5	0.13
Q25	1.0	0.0	0.9	0.3	0.5	0.6	0.7	0.5	0.0	0.0	0.8	0.4	0.00
PEMAT actionability score (%)	90.0	13.7	76.4	28.6	63.1	21.4	59.7	32.1	31.9	23.2	66.7	37.6	0.02

## Discussion

This is the first report introducing a novel breast cancer information-sharing system. The online video platform was peer-reviewed to ensure reliability and was scrutinized by the general public to ensure patient and public involvement. We summarized the quality and viewing status of hereditary breast cancer information currently available on YouTube.

To our knowledge, no reports have focused on the practice of providing medical information on hereditary breast cancer using online video-sharing platforms like YouTube. Several reports analyzed the quality of breast cancer information on YouTube, suggesting the growing awareness of the impact of poor-quality online health-related content [27–29]. Two overseas randomized controlled trials, MAGENTA (Clinicaltrials.gov identifier: NCT02993068) [30] and ProGen (ClinicalTrials.gov identifier: NCT03328091) [31], compared the effectiveness of online genetic education with different counseling approaches. In Japan, one recent report showed the efficacy of smartphone psychotherapy to reduce the fear of cancer recurrence among breast cancer survivors in decentralized randomized controlled clinical trials [32].

Providing medical information using the BC Tube strategy has several advantages. First, we adopted a collaborative format between physicians and citizens and recruited a wide range of volunteers, consisting mainly of non-medical professionals. This collaborative format promotes the delivery of easy-to-understand medical information that reaches a wider audience. Recruiting diverse participants to science fosters fairness and impartiality, and diverse cultural perspectives contribute to better outcomes [33]. Second, the peer review system ensures that the information is evidence-based. Peer review improves the quality and accuracy of scientific research [34] and is an effective and appropriate method. Third, we used the most common online video-sharing platform, YouTube. Viewers can watch the information repeatedly anytime and anywhere. Furthermore, people can share the information with their families, blood relatives, and partners.

About half of the YouTube videos analyzed in this study were from public-interest organizations/companies and these videos obtained more view counts. Public-interest organizations/companies began distributing videos about hereditary breast cancer on YouTube much earlier than other organizations, while most videos from medical institutions and governments were more recent, especially videos about genetic testing and HBOC treatment, which started to be covered by health insurance. Moreover, videos from public-interest organizations/companies and breast cancer survivors acquired more likes and comments, suggesting that videos from non-medical facilities and people received more attention from viewers. On the other hand, the quality of videos from medical institutions and the government was superior. Thus, the popularity did not correspond to the quality of video content. To ensure that high-quality videos are viewed by a wider audience, the posting organization needs to devise ways to improve the promotion and presentation of the videos.

The length of BC Tube videos is limited to less than 10 min to encourage viewers to focus and not move on to other videos. The quality of BC Tube’s content is high, and the likes and view counts show that BC Tube is gaining attention, but needs more recognition. Currently, the videos are played in waiting rooms at some medical facilities and breast clinics to provide breast health information to examinees and patients. In addition, the BC Tube platform is sometimes used for the education of healthcare workers, medical students, and residents at teaching hospitals.

Social media is becoming more diverse, and citizens obtain information from various media platforms, such as Twitter, Instagram, and Facebook, based on their generation and lifestyle. Evaluating information about dissemination methods and modifying the methods in response to changes in citizens and current trends is necessary. In addition, devising a strategy to provide medical information to online-independent citizens is also necessary.

A limitation for this study is that the results of this study depend on YouTube’s search algorithm. On YouTube, efforts are being made to identify information sources based on principles and definitions created by the World Health Organization (WHO) and the National Academy of Medicine (NAM), aiming to provide content originating from reliable sources [35]. In Japan, the YouTube Health team is also working on initiatives to make it easier to identify high-quality information sources [36]. While the findings of this study may not be universal due to the impact of fluctuations in YouTube’s search algorithm, they are important for disseminating higher-quality video information to the public.

In conclusion, we introduced a novel system for providing reliable information on breast cancer using the online video-sharing platform, YouTube, in collaboration with physicians and citizens. Videos distributed by hospitals and governments were of higher quality but need more views or attention. The promotion of online medical information that is reliable, easy-to-understand, and widely recognized is crucial.

## Data Availability

The datasets generated and analyzed in this study are available from the corresponding author upon reasonable request.
